# Theta and Alpha Band Modulations Reflect Error-Related Adjustments in the Auditory Condensation Task

**DOI:** 10.3389/fnhum.2015.00673

**Published:** 2015-12-18

**Authors:** Nikita A. Novikov, Dmitri V. Bryzgalov, Boris V. Chernyshev

**Affiliations:** ^1^Laboratory of Cognitive Psychophysiology, National Research University Higher School of EconomicsMoscow, Russia; ^2^Department of Higher Nervous Activity, Lomonosov Moscow State UniversityMoscow, Russia

**Keywords:** cognitive control, errors, time-frequency decomposition, theta oscillations, alpha oscillations, post-error adaptations, post-error trials

## Abstract

Error commission leads to adaptive adjustments in a number of brain networks that subserve goal-directed behavior, resulting in either enhanced stimulus processing or increased motor threshold depending on the nature of errors committed. Here, we studied these adjustments by analyzing post-error modulations of alpha and theta band activity in the auditory version of the two-choice condensation task, which is highly demanding for sustained attention while involves no inhibition of prepotent responses. Errors were followed by increased frontal midline theta (FMT) activity, as well as by enhanced alpha band suppression in the parietal and the left central regions; parietal alpha suppression correlated with the task performance, left central alpha suppression correlated with the post-error slowing, and FMT increase correlated with both behavioral measures. On post-error correct trials, left-central alpha band suppression started earlier before the response, and the response was followed by weaker FMT activity, as well as by enhanced alpha band suppression distributed over the entire scalp. These findings indicate that several separate neuronal networks are involved in post-error adjustments, including the midfrontal performance monitoring network, the parietal attentional network, and the sensorimotor network. Supposedly, activity within these networks is rapidly modulated after errors, resulting in optimization of their functional state on the subsequent trials, with corresponding changes in behavioral measures.

## Introduction

Successful performance in cognitive tasks critically depends on a number of brain systems supporting, among others, such functions as sustained attention to stimuli, retention and activation of stimulus-response mappings, and inhibition of irrelevant motor responses. A decline in any of these systems may potentially lead to performance errors (van Driel et al., 2012; Navarro-Cebrian et al., 2013). A set of neural processes that keep the activity of these systems at an optimal level is usually described by the term “cognitive control” (Yeung, 2014). Error commission leads to specific adaptations of cognitive control that can be studied in behavioral domain as well as by using psychophysiological methods. In that regard, spectral measures of the electroencephalogram observed after errors may provide helpful insights into the nature of post-error adaptive processes. Among these measures, theta and alpha band power modulations are quite well studied phenomena related to cognitive control functions.

Theta band oscillations (4–7 Hz), which are largely related to the activity in the medial frontal cortex (MFC), were shown to be enhanced after error detection and reward omission (Cavanagh and Frank, 2014; Cohen, 2014). According to the latest evidence, MFC is the main hub of the performance monitoring system that detects situations in which the level of cognitive control should be increased (Cohen et al., 2009b; Womelsdorf et al., 2010a; Cohen and Cavanagh, 2011; Cavanagh et al., 2012; van Driel et al., 2012; Cohen and van Gaal, 2013). Part of the frontal midline theta (FMT) power is believed to reflect the signal of mismatch between the two competing motor representations (Botvinick et al., 2001), or between the actual and the anticipated behavioral outcome (Cohen et al., 2007). Continually growing body of evidence confirms the adaptive role of such theta oscillations. For example, FMT power after error commission was found to positively correlate with post-error accuracy (Cohen and van Gaal, 2013), as well as with post-error slowing of response (PES; Cavanagh and Shackman, 2015).

EEG oscillations in alpha range (8–12 Hz) are believed to reflect the overall level of selective inhibition in neural networks (Klimesch et al., 2007). Specifically, suppression of posterior alpha activity reflects activation of the parieto-occipital network, which largely participates in supporting sensory attention (Klimesch, 2012; Clayton et al., 2015). In the tasks that require high level of sustained attention, posterior alpha power was found to decrease after errors, presumably reflecting the adjustments of attention (Carp and Compton, 2009; Mazaheri et al., 2009; van Driel et al., 2012). Alpha-band suppression at central sites reflects activation of the sensorimotor system, and it was also found to be more pronounced after error commission (Mazaheri et al., 2009). Despite the evidence of the enhanced post-error alpha suppression, its behavioral correlates have not been sufficiently studied (Carp and Compton, 2009).

The current knowledge of brain mechanisms involved in cognitive control is lacking some important pieces of the puzzle. First, error commission may be caused by at least two different mechanisms: (1) failures of motor inhibition, and (2) general attentional lapses related to compromised sensory processing. Accordingly, errors may be followed by different types of post-error adjustments accompanied by their specific electrophysiological correlates (O’Connell et al., 2009a,b; van Driel et al., 2012). Most cognitive control studies use tasks that require inhibition of irrelevant prepotent responses, i.e., they emphasize one specific aspect of cognitive control, namely motor inhibition, while other mechanisms such as those related to attentional lapses remain largely understudied. Second, since the majority of these studies engage visual tasks, it is not clear whether the effects reported (especially those in alpha band) are specific to the visual modality or reflect some general mechanisms of cognitive control. Finally, correct trials that immediately follow erroneous ones were usually investigated in behavioral domain only, while alpha/theta band power modulations occurring on these trials were poorly studied.

Thus, the present study was designed to answer three main questions: (1) whether error-related theta and alpha band power modulations and corresponding behavioral adjustments can be observed in a task that involves no inhibition of prepotent responses; (2) whether these effects, which were well studied in the visual modality, can be found in an auditory task; (3) whether the consequences of post-error adjustments can be observed in spectral characteristics of EEG signal on the next trials following error commissions – i.e., on trials with “post-error correct responses.”

To answer these questions, we recorded EEG when right-handed participants performed an auditory two-choice discrimination task – a version of the condensation task (Posner, 1964). The condensation task well suits the aim of the current study since it produces high cognitive load and requires high level of sustained attention (Chernyshev et al., 2015), but does not imply any prepotent responses to be inhibited or overridden. Furthermore, all stimuli presented in this task are equivalent target stimuli and thus produce no congruency or oddball effects. This allows us to compare correct trials, erroneous trials and post-error correct trials, focusing on specific neural correlates of post-error adaptation.

## Materials and Methods

### Participants and Experimental Conditions

Seventy-one healthy right-handed volunteers with normal or corrected-to-normal vision and normal hearing participated in the present study (mean age 20.1 ± 0.2 years, 18 males). All volunteers reported no history of auditory, neurological, or mental disorders. The experiments were carried out in accordance with the Declaration of Helsinki and its amendments and were approved by the ethics committee of the National Research University Higher School of Economics. Informed consent was signed by each participant before the experiment. All experiments were conducted in a sound-attenuated chamber with a standard ceiling lighting.

### Stimuli

Auditory stimuli were presented to the participants using E-Prime software (Psychology Software Tools, Inc., USA) through a high-quality stereo headset with in-ear design. Four pre-recorded auditory tones were presented. Each tone was a sinusoidal signal of either 500 Hz (‘low’) or 2000 Hz (‘high’), either a pure tone (‘pure’) or the same tone with a broadband noise added to the signal (‘noised’); root mean square amplitude of the noise was -14 dB relative to pure tones. The four stimuli were named in the instruction presented to the participants as (1) ‘low pure,’ (2) ‘low noised,’ (3) ‘high pure,’ and (4) ‘high noised.’ The duration of all stimuli was 40 ms, with rise and fall time 10 ms each; sound pressure level was 95 dB.

### Design and Procedure

An auditory two-choice version of the condensation task was used in the experiment (Chernyshev et al., 2015). The experiment involved six experimental blocks; after the end of each block, participants were offered a short rest.

During each block, a sequence of 100 stimuli was presented; each sequence consisted of four prerecorded audio stimuli (see above) intermixed randomly with equal probability ratio. The stimuli were presented with random stimulus onset asynchrony (SOA) of 2500 ± 500 ms (flat distribution). Visual feedback was given during the experiment: correct responses within the time interval of 300–1700 ms after stimulus onset were reinforced by a ‘smiley’ (a schematic smiling face depicted by eyes and mouth in a yellow circle on a gray background). The rewarding stimulus was presented for 500 ms after correct responses in the center of the screen. Otherwise, the screen remained uniformly gray.

The time interval from the moment of a key pressing until the next auditory stimulus onset was kept to no less than 500 ms by prolonging the particular SOA when needed. The resulting SOA throughout the experiment was 2657 ± 321 ms (M ± SD), with a minimum and maximum of 2063 and 5010 ms correspondingly.

Participants were instructed to hold the gamepad in their dominant (right) hand and to press with a thumb one or the other of the two buttons in response to the stimuli. Participants were also instructed that if he/she would press the correct button after a sound stimulus, a ‘smiley’ would be briefly presented on a screen in front of them.

The participants were offered to familiarize with the following table (**Table 1**), which was given to them printed in a large typeface on a sheet of paper for free viewing and then removed from the chamber before the start of the EEG recording. Table specifies the conjunction contingencies between the two stimulus features (‘high’/‘low’ and ‘pure’/‘noised’) comprising the set of the four stimuli, and the response required to the left and right buttons of the gamepad. Though the rules are very simple, the task cannot be solved at above chance level via processing any single feature but it rather requires a mental conjunction of both features.

**Table 1:** Response contingencies in the experimental task: this table was read as well as handed in printed form to the participants immediately before the experiment.

	High	Low
Pure	Left button	Right button
Noised	Right button	Left button

Before the start of the experimental blocks, the participants were familiarized with the auditory stimuli (the experimenter manually played them to the participants and named them orally (‘low pure,’ ‘low noised’ etc.), and then the participants were blind tested with the stimuli. During this test, all of the participants easily named all of the stimuli correctly, and all of them stated confidently that they could clearly feel the difference between all of the stimuli and knew which button corresponded to each stimulus.

The instruction only informed the participants that they had to press one of two buttons according to the rule specified, but it did not tell them that they had to react as fast as possible, nor did it compel them to make a random choice if they were uncertain. In other words, the instruction did not emphasize time pressure, and participants were implicitly allowed to omit responses.

### Behavioral Data Analysis

In the present study, we used the data from five out of six experimental blocks; the first block was excluded from the analysis, since the participants’ performance was unstable during the initial block as learning progressed. We considered three types of responses: correct responses, errors, and omissions. A response was considered as correct or erroneous if it was committed within the 300–1700 ms time interval after stimulus onset depending on the button pressed; trials with no responses or with responses committed later than 1700 ms were considered as omissions. First, we calculated the percentage of each of these three response types using all trials that contained a response of the corresponding type. Next, we picked out trials belonging to each of the three following conditions: (1) correct responses immediately following correct responses committed on the previous trial – “post-correct correct responses” (cC); (2) errors immediately following correct responses committed on the previous trial – “post-correct erroneous responses” (cE); and (3) correct responses immediately following errors committed on the previous trial – “post-error correct responses” (eC). Trials containing more than one response (double button pressing) were excluded from the analysis at this stage.

All behavioral data analyses were performed within MATLAB software (Mathworks Inc., USA) using custom-made scripts. Response time (RT) for each subject and condition was calculated as the mean response latency over corresponding trials. RT’s of cE and eC trials were compared with RT’s of cC trials using two-tailed paired *t*-test. We also calculated post-error slowing (PES) score for each subject as the ratio between RTs of eC and cC trials. In addition, we calculated correlation between PES and the percentage of errors using rank correlation coefficient (Spearman’s rho).

### Electrophysiological Recording and Analysis

The EEG was recorded using an NVX-52 system (Medical Computer Systems, Russia) with Neocortex Pro software (Neurobotics, Russia) from 27 electrodes in accordance with the modified international 10–10% system and 1 electrooculogram electrode, with a linked earlobe reference and 0.5–200 Hz band-pass filter at a sampling rate of 1000 Hz. Electrode impedance was kept below 10 kΩ for all channels. EEG analysis was performed within MATLAB (MathWorks Inc., USA) software using custom-written scripts and built-in functions of EEGLAB toolbox (Delorme and Makeig, 2004). High-amplitude artifacts exceeding 300 μV were rejected from the data. Signals in bad channels were replaced by spherical interpolations over the neighborhood electrodes. Then 30 Hz low-pass FIR filter was applied to the data, and independent component analysis (ICA) was performed. Independent components related to eye movements were manually selected, and then corresponding components were rejected from the non-filtered data. Finally, we substituted signals in channels contaminated with EMG by spherical interpolation over the neighborhood electrodes; we selected for this procedure those channels, in which the spectral power in 25–45 Hz range exceeded 1.5 standard deviations above the mean value taken over the total number of channels × blocks × subjects in the experimental sample (about 2% of channels × blocks × subjects). In order to reduce volume conduction effects, current source density (CSD) transformation was applied to EEG data using open-source CSD toolbox (Kayser and Tenke, 2006a). CSD transformation can be validly applied to low-resolution EEG data (Kayser and Tenke, 2006b).

Response-locked epochs for each condition (cC, eC, cE) were extracted from the data (-2000 – 2000 ms relative to the response). Individual datasets with less than 10 erroneous epochs were excluded from the analysis at this stage. Thus, the further analysis was performed in 59 participants.

Current source density signal in each channel was translated into time-frequency domain using wavelet transformation using sliding time windows. We used Morlet wavelets with the frequencies ranging from 2 to 40 Hz in steps of 1 Hz; the numbers of cycles were linearly increasing from 2 (on the lowest frequency) to 37.5 (on the highest frequency), thus providing an equal tradeoff between time and frequency resolutions over the whole frequency range. Centers of sliding time windows were uniformly distributed over the interval between -1443 and 1442 ms around the response at 19 ms step. For the further analysis, we used only the frequencies between 4 and 15 Hz, since we were interested in theta and alpha frequencies, and the sliding time windows with centers between -1000 and 1000 ms around the response.

For each time-frequency bin and each electrode, we calculated non-phase-locked spectral power averaged over subsets of trials used for the analysis. First, we calculated the mean total power by averaging squared norms of complex amplitudes over the trials. Next, we calculated phase-locked power by averaging complex-valued amplitudes over the trials, and then taking squared norm of this sum. Non-phase-locked power was calculated as the difference between the total power and the phase-locked power.

Two cross-condition comparisons were performed in our study: cE vs. cC, and eC vs. cC. We used the following RT-matching procedure: within each pair, we used all trials from a condition that was less frequent throughout the experiment (cE or eC), and for each of these trials we selected a matching trial from the other condition (cC) with the closest RT (each trial was used only once). RT-matching helped us to equalize the number of trials between conditions compared, and, consequently, the variance of mean non-phase-locked power estimate, thus avoiding the huge bias in the estimation of the mean difference in non-phase-locked power between conditions. Furthermore, comparison of RT-matched data is likely to reveal the effects related to error commission itself rather than the effects caused by the mean RT difference between conditions. Finally, RT-matching equalizes mean RT across conditions thus allowing to validly compare pre-RT-frequency data between these conditions.

Values of non-phase-locked power for each subject and each condition within a condition pair being compared were organized into 4-D matrix with the following dimensions: rostrality (7 levels), laterality (5 levels), oscillation frequency (12 levels), and time (104 levels). Levels of ‘rostrality’ factor comprised electrode positions from Fp to O (e.g., Fpz, Fz, Fcz, Cz, Cpz, Pz, Oz for the midline). Levels of ‘laterality’ factor comprised the electrode positions from left to right (e.g., T3, C3, Cz, C4, T4 for the central electrodes). Data bins with “spatial” coordinates not corresponding to any electrode were filled with zeroes. Spectral power values were converted to logarithms.

We performed two types of analysis; in the first one, we analyzed event-related spectral perturbations (ERSP) for each of the two conditions within a pair in relation to baseline; in the second one, we analyzed differences in spectral power between conditions. Thus, we report two comparisons between pairs of conditions (cE vs. cC and eC vs. cC), with three datasets for each condition pair (one ERSP map within each of the two conditions itself, and one differential cross-condition map), thus totally producing six data sets.

In order to calculate the baseline, we used stimulus-locked epochs and averaged the spectral power over the time bins with centers in -500–0 ms pre-stimulus time window (independently for each electrode and each frequency). After that, we averaged the resulting pre-stimulus powers over cC and cE conditions, and used the resulting values as a common baseline for all conditions. During the analysis of eC–cC condition pair, we used the baseline calculated for cE–cC pair, because the pre-stimulus interval in eC condition was strongly affected by post-error effects. We used a common baseline for all conditions under comparison because we aimed to focus on post-stimulus effects while avoiding the bias related to pre-stimulus variations caused by aftereffects of the preceding trials.

In within-condition ERSP analysis, we calculated the difference between the logarithm of the spectral power in each data bin and the logarithm of the corresponding baseline power. In cross-condition comparisons, we performed bin-by-bin subtraction of power logarithms between the conditions compared. Both within-condition ERSPs and cross-condition differential 4-D maps calculated for each subject were passed through the same group-level statistical procedure. For each spatial-time-frequency data bin, we looked at the vector of values in this bin obtained for the whole group of subjects, and assessed whether the mean of this vector significantly differed from zero. For ERSP analysis, this is equivalent to comparing the bin with the baseline; for cross-condition analysis, this is equivalent to comparing the corresponding bins between two conditions.

In order to avoid multiple comparison problem, we performed the following statistical procedure. First, we performed a two-tailed paired *t*-test on the group of subjects for each data bin independently, thus producing a 4-D map of *t*-scores. Next, we applied to this map the threshold-free cluster enhancement (TFCE) algorithm (Smith and Nichols, 2009), which results in the map of TFCE-scores of the same dimensionality and size. We used the following parameters of the TFCE algorithm: *E* = 0.5, *H* = 4, 50 threshold levels. Positive and negative *t*-scores were transformed to TFCE scores using two independent runs of the algorithm. After that, we shuﬄed the initial data by flipping the sign of all bins in the map for randomly selected subsets of subjects, and repeated the calculation of TFCE map on this shuﬄed data; this permutational procedure was repeated 1000 times. At each permutation step, we found the maximal (positive) and the minimal (negative) TFCE-score over the entire 4-D map, and then we constructed two distributions: one for the maximal and the other one for the minimal values. Finally, for each bin of the non-shuﬄed TFCE map (independently), we calculated the quantiles of “minimal” and “maximal” distributions the value in this bin falls into, thus obtaining permutation-based *p*-value for this bin. It is important to notice, that the procedure described above provides correction for multiple comparisons simultaneously in the spatial and time-frequency domains, which allowed us to perform time-frequency analysis without introducing any predefined regions of interest (ROIs).

### Correlational Analysis

In order to test the adaptive nature of the effects observed, we performed a correlational analysis. We used the results of the TFCE-based time-frequency analysis described above, and selected three ROIs as continuous aggregations of bins with significant cE–cC spectral power difference within predefined frequency bands of 4–7 Hz (theta band) and 8–12 Hz (alpha band). We calculated correlation coefficients (Spearman’s rho) between PES/total percentage of errors and spectral power differences between cE and cC trials, averaged over the ROIs described above. Correlation analyses for the percentage of errors and for PES were conducted independently; in each analysis, three comparisons (corresponding to three ROIs) were performed. We used the following permutational procedure to meet the problem of multiple comparisons. We randomly shuﬄed the list of the independent variable values, and then we calculated correlation coefficients between this list and the unshuﬄed lists of values of the dependent variables. This procedure was repeated 1000 times; at each step, we chose minimal and maximal values of the three correlation coefficients obtained. Finally, we determined the quantiles of “minimal” and “maximal” distributions the unpermuted coefficients fall into, thus obtaining corrected *p*-values for these coefficients.

In order to confirm and extend in time domain the results of the correlational analysis described above, we additionally analyzed time courses of correlation coefficients. We used the same frequency bands and groups of channels as in the analysis described above (thus, again, using the same three ROIs), but now we calculated correlation coefficients with total percentage of errors and PES for all time bins from 0 to 1000 ms. Six analyses (three ROIs × two behavioral variables) were conducted independently. In each analysis, we calculated a correlation coefficient (Spearman’s rho) between the cE–cC power difference and the corresponding behavioral variable for each time bin. Next, we shuﬄed the list of the independent variable values and recalculated the correlation coefficient for each time bin; this procedure was repeated 1000 times. At each step, we stored the minimal and maximal correlation coefficients over time bins, thus obtaining two distributions of permuted correlation coefficients. These distributions were compared with the unpermuted vector of correlation coefficients, and the vector of corresponding *p*-values (corrected for multiple time bins) was obtained.

All data are presented in the text and in figures as a mean value ± standard deviation.

## Results

### Behavioral Data

Participants made on average of 85.9 ± 9.4% of correct responses, 10.2 ± 7.9% of errors, and 3.8 ± 3.2% of omissions of response. Average RT was 864.4 ± 80.0 ms on cC trials (“post-correct correct responses”), 858.2 ± 92.2 ms on eC trials (“post-error correct responses”), and 975.8 ± 134.2 ms on cE trials (“post-correct erroneous responses”). RT on cE trials was significantly larger than on cC trials (*t* = 9.48, *p* < 0.001). There was no significant RT difference between eC and cC trials (*t* = -0.74, *p* = 0.46). Mean PES was equal to 0.99 ± 0.08. PES did not significantly differ from 1 (*t* = -0.53, *p* = 0.60). No significant correlation between PES and the percentage of errors was found (*r* = -0.15, *p* = 0.21).

### Theta and Alpha Band Power on Erroneous and Post-Error Correct Trials

**FIGURE 1:**
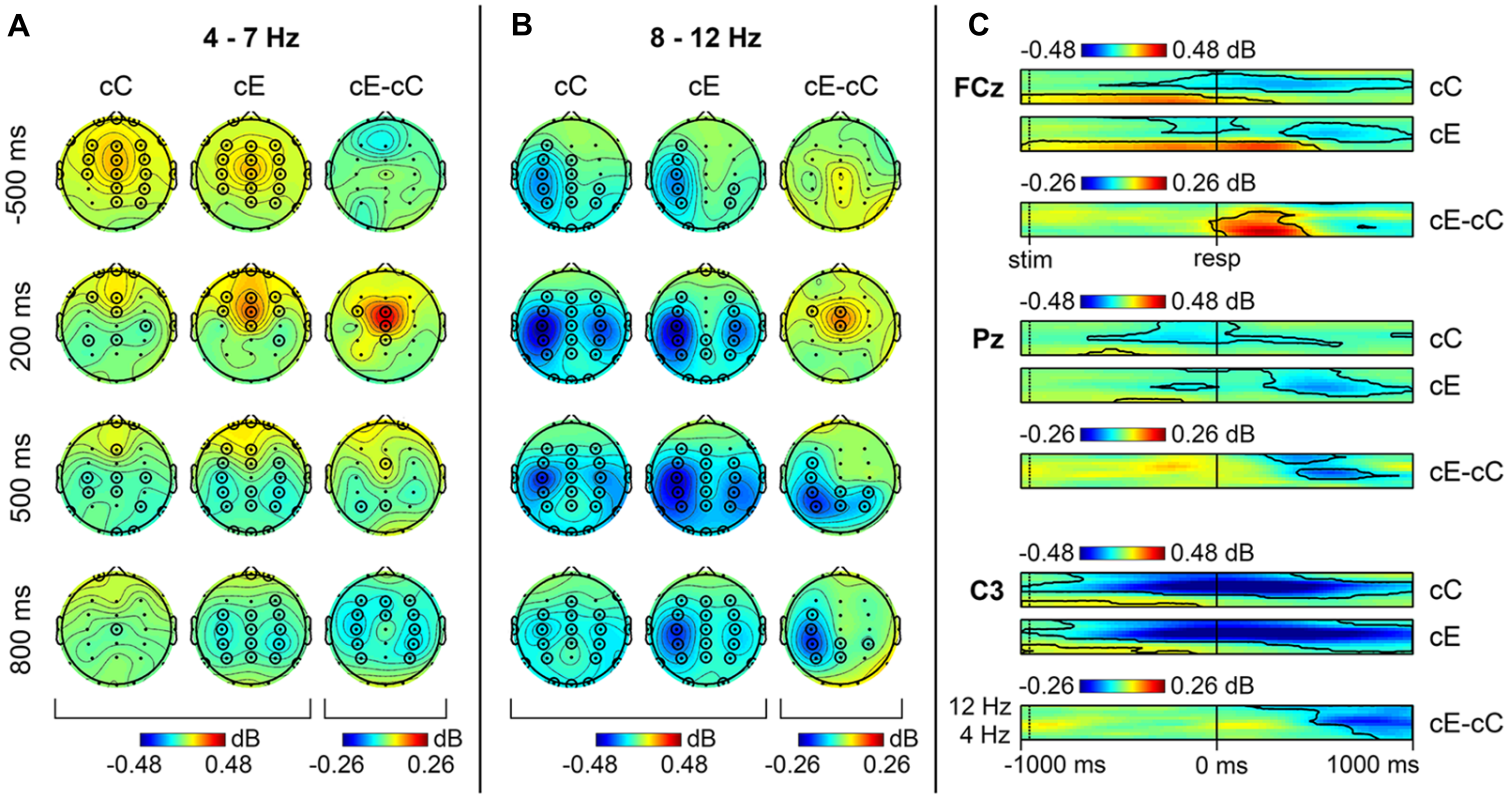
**Non-phase locked oscillatory activity in theta and alpha range on cC and cE trials**. Topographical maps of theta **(A)** and alpha **(B)** band activity at time points relative to the response. Left: spatial distribution of ERSP on cC trials; middle: spatial distribution of event-related spectral perturbations (ERSP) on cE trials; right: spatial distribution of cE–cC log-power difference. Each map is averaged over data bins falling into 50 ms time interval. Significant electrodes (*p* < 0.05, 4-D TFCE) are highlighted by black circles (each electrode is highlighted if at least one time bin is significant within 50 ms interval). **(C)** Time-frequency plots of theta and alpha band activity at FCz, Pz, and C3 electrodes. Black contours show significant areas (*p* < 0.05, 4-D TFCE). Top: dynamics of ERSP on cC trials; middle: dynamics of ERSP on cE trials; bottom: dynamics of cE–cC log-power difference.

The analysis of the non-phase-locked component of the ERSP for cC and cE trials indicated a significant increase in 4–7 Hz theta power relative to the pre-stimulus baseline for these conditions in most channels, excluding the occipital ones (*p* < 0.05, TFCE-based permutational statistics). This activity started soon after the stimulus presentation and quickly terminated after the correct response commission (**Figures 1A,C**). As can be seen in **Figure 1C**, on erroneous cE trials, theta band activity lasted longer until about 400 ms after the response commission, while on correct cC trials the activity in theta range became lower than the baseline shortly after the response. The maximum of theta band power was located in the frontal midline region, slightly moving from central to frontal sites during the trial (**Figure 1A**).

Alpha power (8–12 Hz) on both cC and cE trials was significantly decreased (*p* < 0.05, TFCE-based permutational statistics) relative to the pre-stimulus baseline during the whole time window under analysis (-1000–1000 ms), with its minimum immediately after response commission. This alpha band desynchronization was visible in many central and parietal electrodes, but it was most pronounced at the left central sites before the response and bilaterally over the central regions after the response (**Figure 1B**).

Comparison of erroneous cE trials with RT-matched correct cC trials revealed that non-phase-locked theta power (4–7 Hz) was significantly larger (*p* < 0.05, TFCE-based permutational statistics) in 0–400 ms post-RT window after errors than after correct responses, as can be seen in **Figure 1C**; this increase was localized in the frontal midline region (**Figure 1A**).

Non-phase-locked alpha power (8–12 Hz) was significantly decreased (*p* < 0.05, TFCE-based permutational statistics) after erroneous cE responses compared with correct cC ones. As can be seen in **Figure 1C**, this decrease started within 400 ms after the response and lasted until the end of the analysis window (1000 ms). Initially it was more pronounced at posterior parietal sites, and then moved to left central regions (**Figure 1B**).

**FIGURE 2:**
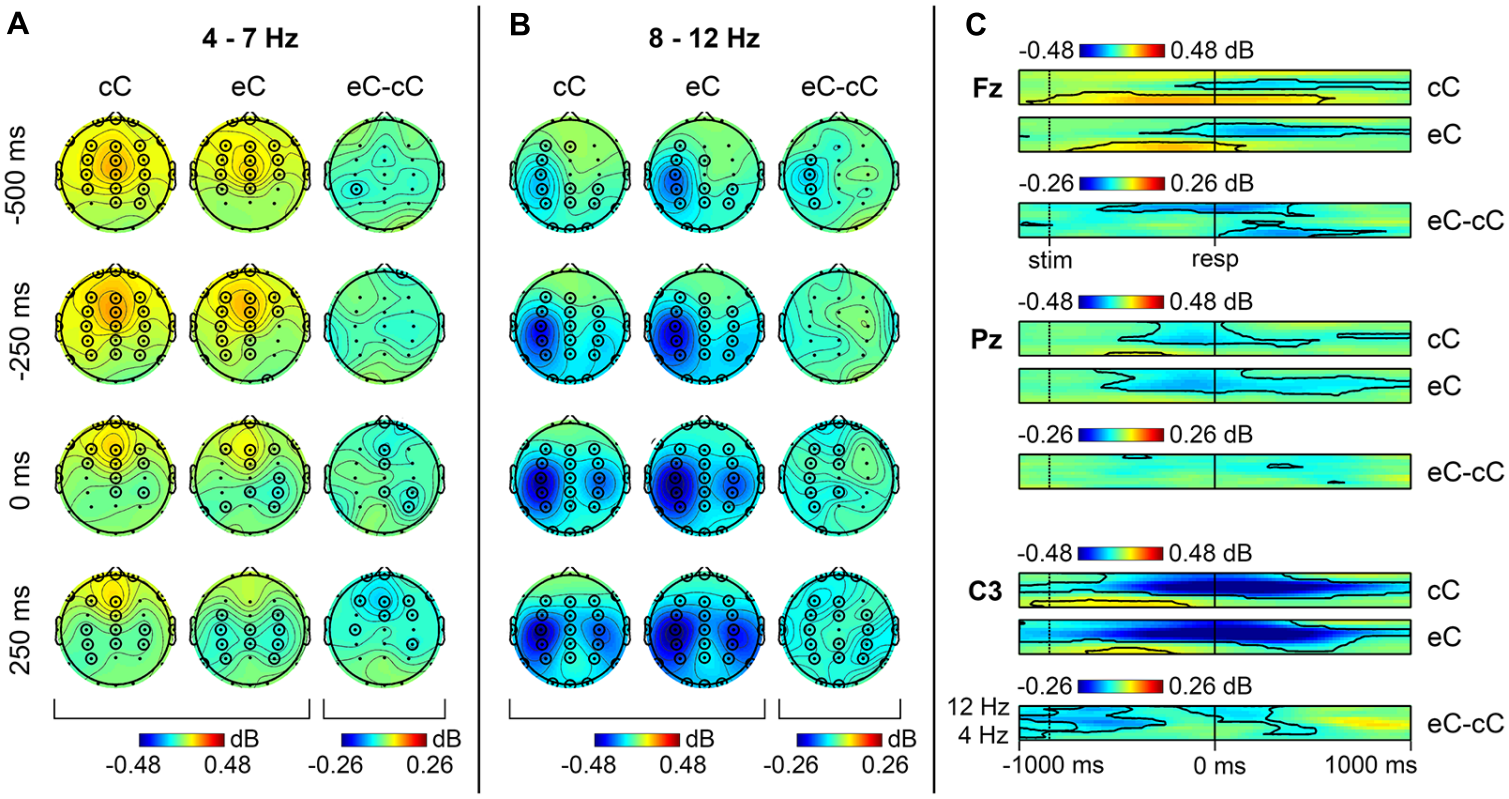
**Non-phase locked oscillatory activity in theta and alpha range on cC and eC trials**. Topographical maps of theta **(A)** and alpha **(B)** band activity at time points relative to the response. Left: spatial distribution of ERSP on cC trials; middle: spatial distribution of ERSP on eC trials; right: spatial distribution of eC–cC log-power difference. **(C)** Time-frequency plots of theta and alpha band activity at Fz, Pz, and C3 electrodes. Top: dynamics of ERSP on cC trials; middle: dynamics of ERSP on eC trials; bottom: dynamics of eC–cC log-power difference. Other conventions as in **Figure 1**.

Similar to cE and cC conditions, non-phase locked component of ERSP in theta band on post-error correct trials (eC) demonstrated a significant increase around the response. Again, the maximum of theta band power was located at frontal sites (**Figure 2A**). However, theta band activity on eC trials terminated earlier than on cC trials (**Figure 2C**). Non-phase locked component of ERSP in alpha range showed a significant suppression widely distributed over the scalp, compared with the baseline (**Figures 2B,C**).

As can be seen in **Figure 2C**, comparison of post-error eC trials with RT-matched correct cC trials revealed a significant decrease (*p* < 0.05, TFCE-based permutational statistics) of non-phase-locked theta power in 0–600 ms post-RT window on post-error correct eC trials compared with correct cC trials; this decrease was most pronounced at frontal midline sites (**Figure 2A**).

A significant decrease (*p* < 0.05, TFCE-based permutational statistics) of non-phase-locked alpha power at the left central sites on eC compared with cC trials was observed (**Figure 2B**); as can be seen in **Figure 2C**, it started at the beginning of the analysis window (1000 ms before the response) and lasted till 250 ms before the response. Another significant decrease (*p* < 0.05, TFCE-based permutational statistics) of non-phase-locked alpha power on eC trials compared with cC trials was observed in -150–500 ms time window around the response (**Figure 2C**); it started in the left hemisphere and then spread over the whole scalp after the response commission (**Figure 2B**).

### Correlations Between Theta and Alpha Band Power and Behavioral Measures

In order to examine the relationships between error-related theta and alpha band power modulations and corresponding behavioral measures, we performed correlational analysis between cE–cC spectral power differences and total percentage of errors/PES. Based on the results of the TFCE-based time-frequency analysis, we selected the following ROI’s for the correlational analysis: (1) ROI1: 4–7 Hz, 0–400 ms, FCz + Cz; (2) ROI2: 8–12 Hz, 400–700 ms, CP3 + CPz + CP4 + P3 + Pz + P4; (3) ROI3: 8–12 Hz, 500–1000 ms, FC3 + C3 + CP3.

**FIGURE 3:**
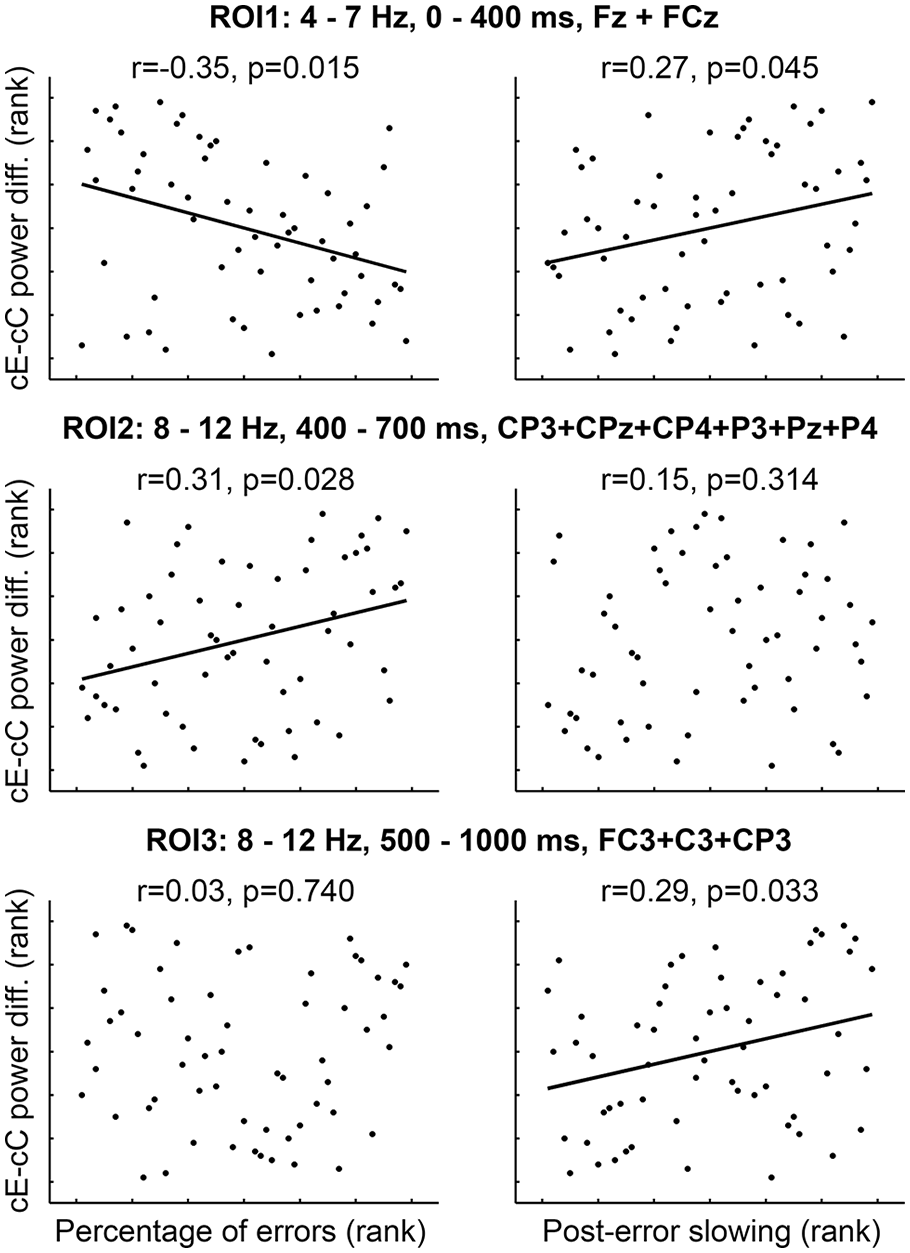
**Rank correlations (Spearman’s rho) between behavioral measures and cE–cC power difference averaged over spatial-time-frequency ROI’s**. *p*-values corrected for multiple comparisons (three ROI’s) using permutation-based test (see Materials and Methods).

The percentage of errors negatively correlated with the difference in EEG spectral power between cE and cC conditions in midline theta ROI1 (*r* = -0.35, *p* = 0.015; **Figure 3**, left column, top panel) and positively correlated with the difference in parietal alpha ROI2 (*r* = 0.31, *p* = 0.028; **Figure 3**, left column, middle panel). PES positively correlated with the EEG spectral power difference between cE and cC conditions in midline theta ROI1 (*r* = 0.27, *p* = 0.045; **Figure 3**, right column, top panel) and left-central alpha ROI3 (*r* = 0.29, *p* = 0.033; **Figure 3**, right column, bottom panel). All other correlations were non-significant (**Figure 3**).

**FIGURE 4:**
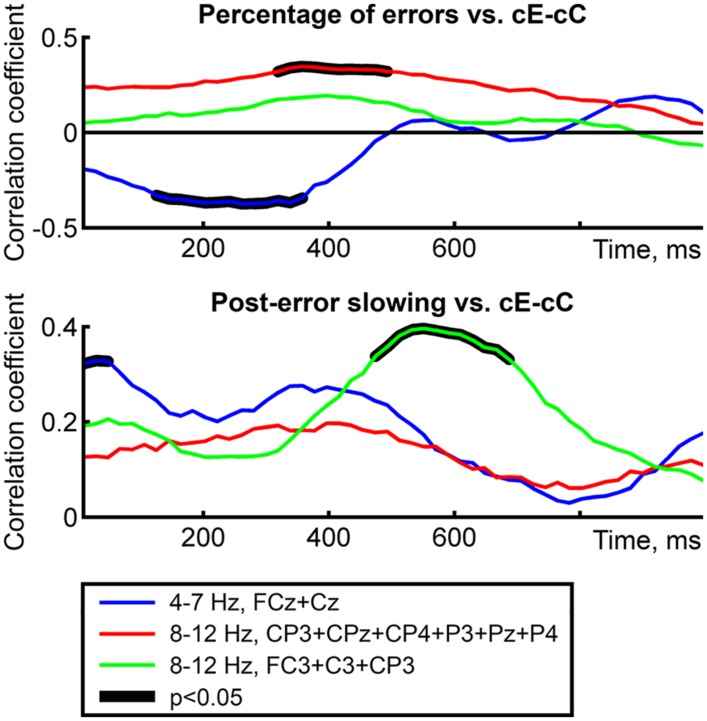
**Time courses of rank correlations (Spearman’s rho) between behavioral measures and cE–cC power difference averaged over spatial-frequency ROI’s**. Top and bottom panels correspond to two behavioral variables; line color represents ROI’s. Thick black lines represent intervals of significant correlations (*p* < 0.05, permutation-based correction for multiple time bins).

In general, the analysis of correlation coefficient time courses confirmed the results presented above (**Figure 4**). Negative correlation between cE–cC theta power difference and percentage of errors was significant in 120–360 ms time window; positive correlation between parietal cE–cC alpha power difference and percentage of errors was significant in 320–490 ms time window; positive correlation between cE–cC theta power difference and PES was significant in 0–65 ms time window; positive correlation between left central cE–cC alpha-band power difference and PES was significant in 470–690 ms time window. As it can be seen in **Figure 4**, time windows of significant correlations largely overlapped with the time windows in which significant cE–cC power difference was observed.

## Discussion

### Summary of Results

In the present study, we examined error-related changes in theta and alpha bands and corresponding behavioral adjustments under the auditory version of the condensation task. In addition to erroneous trials, we investigated correct trials immediately following errors.

At group level, errors were committed slower than correct responses, while PES was not statistically significant at group level. In addition, PES did not correlate with the total error rate.

Time-frequency analysis revealed that performance errors led to a burst of the FMT power and to enhanced alpha suppression at parietal and left central sites in a prolonged time window after response commission. Stronger post-error FMT increase was related both to better task performance (reduction in the percentage of errors) and to stronger PES. More pronounced post-error parietal alpha suppression was associated with better task performance (reduction in the percentage of errors), while less pronounced post-error left central alpha-band suppression was associated with stronger PES.

Comparison of correct trials following erroneous trials (eC) and correct trials following correct trials (cC) revealed decreased post-response FMT power and a general drop in alpha band power, mostly pronounced in the left central regions between the stimulus and the response; the latter effect spread over the entire scalp closer to the time of response commission.

### Behavioral Indices of Lost and Regained Cognitive Control

In the current study, we observed longer RTs for erroneous responses compared to correct ones. Most tasks used in cognitive control studies (including the SART, Simon task, flanker task, etc.) require overriding some prepotent (“automatic”) responses, and participants are usually instructed to respond as fast as possible. In such tasks, probability of error commission is higher during spontaneous decreases of the motor threshold, thus leading to error speeding (Ridderinkhof, 2002; Ratcliff and McKoon, 2008; Dudschig and Jentzsch, 2009). In contrast, the condensation task used in our experiment involves no obvious prepotent responses one needs to override; at the same time, this task is demanding for the activity of stimulus-processing systems because complex bivalent stimuli and non-intuitive stimulus-to-response mapping are used. Errors in complex or accuracy demanding tasks tend to occur in situations of decision uncertainty, which leads to slowing of erroneous responses (Wilding, 1971; Dyson and Quinlan, 2003; Ratcliff and McKoon, 2008; O’Connell et al., 2009b; van Driel et al., 2012; Cohen and van Gaal, 2013). In agreement with this logic, RT of errors in our task was predictably larger than RT of correct responses.

In our study, we did not observe any significant PES effect at group level. This can be explained by the presence of two different effects that push the RT in opposite directions. Generally, error-related adaptations of cognitive control fall into two major groups: (a) a non-specific increase of motor threshold (“proactive” strategy), and (b) a specific enhancement of task-relevant information processing (“preemptive” strategy; Ridderinkhof, 2002; Ridderinkhof et al., 2011). The motor threshold increase is related to PES (Dudschig and Jentzsch, 2009; Cohen, 2014), while the specific adaptation may presumably lead to the RT decrease due to enhanced stimulus processing (Cavanagh and Frank, 2014). It was shown that intensity of specific and non-specific adaptations may differ between subjects (King et al., 2010), the fact that can explain the absence of a group-level PES effect in our task. We found no correlation between PES and the percentage of errors; this suggests that neither of the two strategies was more successful than the other – the finding that stays in line with other studies (e.g., Danielmeier and Ullsperger, 2011). The absence of a significant PES can be also explained by the fact that our task implies no fast prepotent responses and does not involve any strong time pressure. In such situation, participants may have had enough time to slow down during the erroneous trial itself rather than on the following one.

### Theta Band Oscillations on Erroneous Trials and on Correct Trials Immediately Following Errors

Frontal midline theta modulation following errors and reward omissions is known to be closely related to the event-related potential components such as error-related negativity (ERN) and feedback-related negativity (FRN) that fall into theta frequency band and have similar localization in MFC (Cavanagh et al., 2012). However, post-error modulation of non-phase-locked component of FMT was also observed experimentally (Trujillo and Allen, 2007; Cohen and Donner, 2013). In the present study, we focused on this non-phase locked FMT activity to be sure that the effects observed are not related to the event-related potentials evoked by feedback presentation.

Theta band power underwent a strong and widespread increase with its maximum at the frontal midline: it started soon after stimulus presentation and lasted until the behavioral response. Such theta band activity is believed to support coordination of various task-related neural processes (sensory processing, memory retrieval, activation of rule representations, initiation of motor program, etc.), with MFC being the central hub for this integration (Womelsdorf et al., 2010b; Cavanagh and Frank, 2014).

Unlike correct responses, performance errors in our task were followed by a much longer-lasting power increase in theta range extending beyond the RT; this resulted in a large difference in non-phase-locked theta power between cE and cC trials within the post-response time window. In contrast to a more widespread ERSP increase in theta band, this differential theta band activity was confined to a narrow frontal midline region, supporting the view that MFC is the main area related to detection of errors and negative outcomes (Cavanagh and Frank, 2014; Yeung, 2014).

On correct trials following errors (eC), post-response theta band activity subsided earlier than on cC trials, leading to a significant negative difference in non-phase-locked theta power between these types of trials in the post-response time window. This difference had frontal localization with the largest effect at frontal midline sites, thus being an inverse of the increase in theta activity observed after error commission, within a similar post-stimulus time range.

While post-error FMT increase stays well in accordance with a huge body of cognitive control studies using a variety of tasks (Mazaheri et al., 2009; Womelsdorf et al., 2010a; Cohen and van Gaal, 2013; Cavanagh and Shackman, 2015), the decrease in post-response FMT power on correct trials following errors, to our knowledge, has never been demonstrated before.

Frontal midline theta power is known to be increased in situations that require readjustment of certain neural processes in order to provide proper task performance; these situations include novelty, conflict, punishment, and error detection (Cavanagh and Frank, 2014). The increase in FMT power after error commission may be partially explained within the conflict monitoring theory. It holds that erroneous trials are accompanied by a high level of conflict between the motor program associated with the produced erroneous response and the motor program associated with the intended correct response (Yeung et al., 2004); this conflict is detected by MFC, resulting in the increased FMT power (Cavanagh et al., 2009; Cavanagh and Frank, 2014). FMT modulations caused by negative feedback are mostly studied within the framework of the reinforcement learning theory. It states that FMT is increased after detection of any discrepancy between the actual and the anticipated rewards, which is also a function of the MFC (Cohen et al., 2007, 2009a; Christie and Tata, 2009; van de Vijver et al., 2011). Despite the fact that error- and reward-related processes, probably, cannot be fully unified within a single theoretical framework (Yeung and Nieuwenhuis, 2009; Alexander and Brown, 2011), they both lead to an increase in FMT power.

In accordance with the literature cited above, post-error theta burst observed in our task may reflect two complementary processes occurring in MFC through functioning of two distinct but largely overlapping neural networks: (1) detection of the conflict between correct and erroneous motor programs, and (2) detection of the mismatch between the anticipated and the actual behavioral outcome (positive feedback was anticipated by the subject, but was not received).

The decrease in post-response FMT power on eC trials compared with cC trials can be explained using similar logic. First, post-error adaptation of cognitive control leads to stronger activation of the correct motor program compared with the incorrect one, thus producing weaker response conflict, and, thus, less pronounced MFC activation. Second, in the state of increased cognitive control, decision certainty in a subsequent correct outcome may increase. Consequently, the mismatch between the actual outcome (produced by the positive feedback) and the anticipated outcome (related to the certainty that the response produced was correct) is decreased, leading to decreased FMT power.

### Alpha Band Oscillations on Erroneous Trials and on Correct Trials Immediately Following Errors

We observed a widespread central-posterior alpha band desynchronization in the whole analysis time window (-1000–1000 ms relative to the response) for all conditions. It was most pronounced over the left central regions before the response, with an additional weaker focus of alpha band desynchronization in the right central regions after response commission. Alpha band activity is known to reflect excitatory-inhibitory balance in cortical structures, with stronger oscillations corresponding to less active state (Klimesch et al., 2007). Oscillations in alpha range over the central areas are usually referred to as mu rhythm (Pfurtscheller and Lopes da Silva, 1999). For hand movements, mu desynchronization starts in the contralateral hemisphere during movement preparation and becomes bilateral just before the movement onset (Szurhaj et al., 2001); our findings stay in agreement with this pattern (note, that the effect observed in our study was most evident on the left side, while all participants responded with their right hand). Some authors suggest that sensorimotor activation and mu rhythm suppression may be related not only to action preparation, but also to a more general decision-making process, including evidence integration (Pineda, 2005; Tosoni et al., 2014).

In the present study, alpha band suppression lasted longer after errors than after correct responses, leading to a negative alpha band power difference between cE and cC trials observed in the post-response time window (400–1000 ms). In the earlier part of this time window (400–700 ms), subtraction of cC from cE revealed a negative alpha band power difference over the parietal regions. Toward the end of this time window, the scalp distribution of alpha band power difference became similar to the scalp distribution of the ERSPs for both correct and erroneous trials, with its maximum over the left central region.

A very similar pattern of post-response alpha band power difference between erroneous and correct trials was observed by Mazaheri et al. (2009) using SART. In that study, negative difference in alpha band between erroneous and correct trials was distributed over posterior and central sites in 100–800 ms time window, and concentrated in the central regions between 500 and 1000 ms after the response.

Differential alpha band effect in the later window was localized in the sensorimotor areas, and it was interpreted by Mazaheri et al. (2009) as the adaptive engagement of these areas supporting increased inhibitory motor control in future. Given the possible role of the sensorimotor system in decision-making, the same interpretation can also be applied to our results (Pineda, 2005). However, we cannot exclude a possibility that some part of differential post-error mu suppression does not bear an adaptive nature (in the strict sense of this word) and reflects continuation of stimulus processing in the uncertain situation, or covert re-selection of the correct response that does not lead to the actual movement commission.

Post-error posterior alpha suppression in the early post-response time was observed by van Driel et al. (2012) in 150–500 ms time window at occipital-parietal sites in SART and in the version of the Simon task predisposing to attentional lapses. Interestingly, this effect was not found in the version of the Simon task predisposing to motor inhibition failures, thus further confirming the role of alpha suppression in adaptive attentional modulations (van Driel et al., 2012). Similar alpha band power difference reported by Mazaheri et al. (2009) was localized in the posterior cortex, thus suggesting its role in attentional modulation. Decreased post-error alpha band power (most pronounced at parietal sites) was also observed in the Stroop and flanker tasks (Carp and Compton, 2009; Compton et al., 2014), the findings that were interpreted by the authors in terms of greater cortical arousal produced by error commission. Posterior cortex presumably contains overlapping networks that support attention in different modalities (Lee et al., 2014). Auditory attention modulates both BOLD-signal in the parietal cortex (Hill and Miller, 2010) and the power of posterior alpha activity (Kerlin et al., 2010; Banerjee et al., 2011; Ahveninen et al., 2013). Thus, our data suggest that the findings of post-error suppression of posterior alpha activity obtained in visual tasks can be extended to the auditory modality; it is likely to reflect the signal of adaptive attentional modulation that has common manifestations during both visual and auditory tasks.

It is important to mention that alpha band power difference observed in our study cannot be explained by processing the visual feedback stimulus that was presented after correct responses but omitted after errors. Indeed, in this case, stronger alpha suppression should have occurred on cC trials rather than on cE trials; such effect was indeed visible at occipital electrodes, but it was not significant.

On post-error correct trials (eC), we found more pronounced alpha-band suppression compared with cC trials. This differential effect was observed in -1000 – -250 ms pre-response time window in the left sensorimotor regions (reflecting the fact that sensorimotor activation ramps up faster on eC compared with cC trials); in –150–150 ms time window it was distributed over the whole left hemisphere, and in 200–500 ms window it was spread over the entire scalp. We interpret this effect as a correlate of increased engagement of the sensorimotor system, presumably supporting more accurate decision-making and response selection. Interestingly, eC–cC difference in the late post-response window (500–1000 ms) was positive over the left central regions (**Figure 2C**), which inversely mirrors the situation observed for erroneous trials; however, this effect did not reach statistical significance.

### Relations Between Post-Error Theta and Alpha Band Modulations and Behavior

Errors may lead to a number of behavioral adjustments that are observed on subsequent trials and consist of modulations of RT or accuracy (Danielmeier and Ullsperger, 2011). These modulations are related to post-error changes in activity of particular brain networks and, consequently, can be detected using electrophysiological techniques. We were interested in relations between post-error alpha/theta band power changes and such behavioral measures as PES and total percentage of errors.

Post-error FMT increase is believed to reflect the need of increased cognitive control detected by MFC, and to support the basis of functional connections between MFC and the downstream sensory/motor/modulatory brain regions, where specific adjustments should be made (Botvinick et al., 2001; Narayanan et al., 2013; Cavanagh and Frank, 2014). Consequently, both FMT power and ERN/FRN amplitude, which is closely related to FMT power, often correlate with the intensity of corresponding behavioral adaptations. Specifically, stronger FMT power/larger ERN amplitude are associated with more pronounced PES (Cavanagh and Shackman, 2015) and with better post-error accuracy (Carp and Compton, 2009; Themanson et al., 2012; Cohen and van Gaal, 2013). Our data stay in line with these observations.

In our study, lower total percentage of errors (i.e., better performance) was related to stronger post-error FMT burst and to more pronounced post-error alpha suppression in parietal regions. FMT burst and the following posterior alpha suppression are likely to reflect two stages of one and the same sequence of events that starts from error detection (directly or via the absence of the positive feedback) and leads to adaptive attentional modulation. Direct evidence for correlation between FMT increase and posterior alpha decrease was found by Mazaheri et al. (2009) on trial-by-trial basis; indirect evidence comes from Cohen and van Gaal (2013), who demonstrated increased coherence between mid-frontal and posterior sites in theta range after errors. Given the fact that condensation task used in the current study critically depends on the level of sustained attention, it seems reasonable that subjects with stronger post-error attentional adjustments demonstrate better performance.

We also found in the current study that subjects who demonstrated stronger PES had more pronounced post-error midfrontal FMT increase and less pronounced post-error alpha band suppression in the sensorimotor regions. It is believed that MFC can downregulate motor cortex after error commission via activation of an inhibitory network (Danielmeier and Ullsperger, 2011), and this process may be supported by increased post-error coupling in theta band between midfrontal and sensorimotor areas (Narayanan et al., 2013). Thus, PES-related post-error FMT increase observed in our task is likely to reflect the detection of the need for increasing motor threshold, while smaller post-error sensorimotor alpha band suppression may reflect the influence on the sensorimotor cortex produced by the motor inhibition network.

Post-error FMT increase significantly correlated with PES and the percentage of errors within differing time windows (0–65 and 120–360 ms, respectively), presumably suggesting that PES is more sensitive to response conflict rather than to detection of feedback omission. Correlation between the posterior alpha power difference and the percentage of errors was observed in the similar (but slightly earlier) time window than the between-condition effect itself (320–490 and 400–700 ms, respectively). One possible explanation is that these analyses reveal different aspects of post-error modulation of the parietal network, among which the earlier one is related to good performance but is too subtle to be significant in between-condition analysis. Correlation between sensorimotor alpha suppression and PES was observed in 470–690 ms window, while the suppression itself lasted until the end of the epoch. This discrepancy, together with the fact that post-error sensorimotor alpha suppression was clearly observed in our study, while PES was absent at group level, leads to the possibility that PES-related inhibitory signal is superimposed over some other activity in the sensorimotor system such as stimulus reprocessing or rule reexamination. Sensorimotor alpha suppression did not correlate with the percentage of errors, in agreement with the fact that PES does not strongly affect performance in the condensation task (no correlation between PES and the percentage of errors). On the other hand, parietal alpha suppression did not correlate with PES, supporting the view that specific and non-specific post-error adaptations are produced by relatively independent brain networks (King et al., 2010).

## Conclusion

In this study, we aimed to describe error-related modulations of theta and alpha band activity in the auditory version of the condensation task, as well as to specify relations between these modulations and behavioral measures of the task performance.

We found the post-error FMT power increase, which positively correlated with both PES and successful performance (supposedly, reflecting error detection). It was followed by the posterior partial alpha suppression, which was related to good performance (probably, reflecting attentional reconfiguration). Then, the sensorimotor alpha band suppression was observed, which was less pronounced for subjects with stronger PES (presumably, reflecting stimulus reprocessing with superimposed signal from motor inhibition system). On a subsequent post-error trial, sensorimotor alpha band suppression ramped up faster, alpha suppression distributed over the entire scalp was observed after a response, and the post-response FMT power was reduced, reflecting jointly the state of increased cognitive control.

Current findings extend the current knowledge concerning error-related behavioral adjustments and corresponding modulations of theta and alpha band activity to a task that involves no inhibition of prepotent responses. Moreover, our results extend this knowledge to the auditory modality. Additionally, we have demonstrated that the consequences of post-error adjustments can be observed in theta and alpha band oscillations on trials with “post-error correct responses” – i.e., they have longer-lasting effects visible during the following correct trial.

In summary, our results confirm the functional role of theta and alpha band oscillations in cognitive control and suggest that at least three brain networks exhibit error-related activity: the medial prefrontal network (monitoring the need for increased cognitive control), the parietal attentional network (supporting sustained attention), and the sensorimotor network (decision making and action selection). Further research is needed to examine the correlational and causal relationships between these systems as well as the specific role of each of the systems in post-error behavioral improvement.

## Conflict of Interest Statement

The authors declare that the research was conducted in the absence of any commercial or financial relationships that could be construed as a potential conflict of interest.
